# Hydrothermal Synthesis and Properties of Yb^3+^/Tm^3+^ Doped Sr_2_LaF_7_ Upconversion Nanoparticles

**DOI:** 10.3390/nano13010030

**Published:** 2022-12-21

**Authors:** Bojana Milićević, Jovana Periša, Zoran Ristić, Katarina Milenković, Željka Antić, Krisjanis Smits, Meldra Kemere, Kaspars Vitols, Anatolijs Sarakovskis, Miroslav D. Dramićanin

**Affiliations:** 1 Centre of Excellence for Photoconversion, Vinča Insitute of Nuclear Sciences—National Institute of the Republic of Serbia, University of Belgrade, P.O. Box 522, 11001 Belgrade, Serbia; 2 Institute of Solid State Physics, University of Latvia, Kengaraga Street 8, LV-1063 Riga, Latvia

**Keywords:** nanophosphor, fluoride, morphology, upconversion, Tm^3+^ emission, NIR emission

## Abstract

We report the procedure for hydrothermal synthesis of ultrasmall Yb^3+^/Tm^3+^ co-doped Sr_2_LaF_7_ (SLF) upconversion phosphors. These phosphors were synthesized by varying the concentrations of Yb^3+^ (x = 10, 15, 20, and 25 mol%) and Tm^3+^ (y = 0.75, 1, 2, and 3 mol%) with the aim to analyze their emissions in the near IR spectral range. According to the detailed structural analysis, Yb^3+^ and Tm^3+^ occupy the La^3+^ sites in the SLF host. The addition of Yb^3+^/Tm^3+^ ions has a huge impact on the lattice constant, particle size, and PL emission properties of the synthesized SLF nanophosphor. The results show that the optimal dopant concentrations for upconversion luminescence of Yb^3+^/Tm^3+^ co-doped SLF are 20 mol% Yb^3+^ and 1 mol% Tm^3+^ with EDTA as the chelating agent. Under 980 nm light excitation, a strong upconversion emission of Tm^3+^ ions around 800 nm was achieved. In addition, the experimental photoluminescence lifetime of Tm^3+^ emission in the SLF host is reported. This study discovered that efficient near IR emission from ultrasmall Yb^3+^/Tm^3+^ co-doped SLF phosphors may have potential applications in the fields of fluorescent labels in bioimaging and security applications.

## 1. Introduction

Upconversion (UC) phosphor materials doped or co-doped with trivalent lanthanide (Ln^3+^) ions have drawn considerable attention, since four-electron configurations of Ln^3+^ ions should split by electron–electron repulsion and spin–orbit coupling, resulting in a rich energy-level pattern that can be easily populated by the near infrared (NIR) laser source [1,2]. Under a NIR laser source, UC phosphors effectively convert energy into shorter wavelength emissions (NIR, visible, or ultraviolet) via multiphoton absorption or efficient energy transfer [3]. These unique features of Ln^3+^-doped/co-doped UC phosphors enable a wide range of applications, such as LED devices [4], solar energy conversion [5], temperature sensors [6,7,8], latent fingerprint detection [9,10], biomedical imaging [11,12], food safety detection [13], etc. The NIR-to-NIR UC luminescence mechanism is gaining popularity due to its efficient way of producing NIR emission at nearly 800 nm when excited by a commercially available laser source (~980 nm) [9,14,15,16,17,18,19,20,21]. It is well known that the Yb^3+^ ion, with its simple energy level structure (ground state ^2^F_7/2_ and excited state ^2^F_5/2_), strong absorption band in the wavelength range of 860–1060 nm, and relatively long luminescence lifetime (1–2 ms), is an excellent sensitizer for energy transfer to RE ions [21,22,23]. Ln^3+^ ions such as Pr^3+^, Er^3+^, Tm^3+^, and Ho^3+^ have been recognized as co-activators in Yb^3+^/Ln^3+^ UC phosphors due to the position of their energy levels and the possibility of efficient radiative transitions under NIR light sources [9,14,15,16,17,18,19,20,21,22,23,24]. Among them, Yb^3+^/Tm^3+^ UC phosphors have been intensively studied because NIR-to-NIR UC emission is crucial for biomedical imaging [17,18,19,20], security printing applications [15,16] and latent fingerprint detection [9].

The UC luminescence mechanism has been explored in a variety of host materials, including chlorides, fluorides, oxides, vanadates, and others. The selection of appropriate host materials with low phonon energy frequencies to prevent non-radiative relaxation processes and thus improve emission efficiency is essential for UC luminescence. Chlorides have low phonon frequencies (≤300 cm^−1^) and poor chemical stability, which limits their application possibilities, whereas oxide host materials have relatively high phonon frequencies (>500 cm^−1^) and excellent chemical stability [25]. Fluoride materials are thus ideal hosts for UC luminescence due to their low phonon frequency (from 300 to 500 cm^−1^), good chemical stability, and simplicity of dispersion in colloidal form with water or various nonpolar solvents [25,26].

Even though alkaline-Ln^3+^ tetrafluoride phosphors (ALnF_4_, A = Na, K, Li) are the most preferred for efficient UC luminescence, their agglomeration limits applications that require nanoparticles, such as in biomedical imaging [27,28,29,30]. According to several recent studies, alkali–earth–Ln^3+^ nanophosphors (M_2_LnF_7_, M = Ca, Sr, Ba; Ln^3+^ = Y, La, Gd, Lu) are small enough for biomedical imaging applications while exhibiting extremely high UC luminescence [31,32,33,34]. Under 980 nm laser stimulation, Xie et al. observed efficient visible emission of Sr_2_LaF_7_:Yb^3+^, Er^3+^ UC nanophosphors with average particles of around 25 nm [34]. Guo et al. reported that Sr_2_GdF_7_:Er^3+^, Yb^3+^ nanocrystals incorporated into electrospun fibers promote energy transfer processes from Yb^3+^ to Er^3+^, which is crucial for potential applications in the field of noncontact biomedical temperature sensors [8]. In this work, a set of Sr_2_La_1-x-y_F_7_ phosphors with different concentrations of Yb^3+^ (x = 10, 15, 20, and 25 mol%) and Tm^3+^ (y = 0.75, 1, 2, and 3 mol%) ions with respect to La^3+^ ions are prepared hydrothermally at 180 °C. The room temperature photoluminescence spectra of SLF:Yb, Tm under 980 nm excitation clearly show intense NIR emissions in the wavelength range from 750 to 850 nm, with the highest intensity around 800 nm.

Herein, we propose a procedure for the hydrothermal synthesis of small Yb/Tm activated SFL nanoparticles. Further, we documented their NIR-to-NIR UC. This UC process has been given much less attention in Yb^3+^ and Tm^3+^ co-doped phosphors than blue and deep-red UC emissions, although it can considerably expand the fields of application of UC nanophosphors, especially as a suitable fluorescent marker in the development of latent fingerprints. 

## 2. Materials and Methods

### 2.1. Chemicals

Strontium nitrate (Sr(NO_3_)_2_, Alfa Aesar Karlsruhe, Germany, 99%), lanthanum (III) nitrate hexahydrate (La(NO_3_)_3_⋅6H_2_O, Alfa Aesar, 99.99%), ytterbium (III) nitrate hexahydrate (Yb(NO_3_)_3_⋅5H_2_O, Alfa Aesar, 99.9%), thulium (III) nitrate hexahydrate (Tm(NO_3_)_3_⋅6H_2_O, Alfa Aesar, 99.9%), disodium ethylendiaminetetraacetate dihydrate (EDTA-2Na, C_10_H_14_N_2_O_8_Na_2_∙2H_2_O, Kemika, Zagreb, Croatia, 99%), ammonium fluoride (NH_4_F, Alfa Aesar, 98%), 25% ammonium solution (NH_4_OH, Fisher, Loughborough, Leicestershire, United Kingdom) and de-ionized water were used as starting materials without further purification.

### 2.2. Synthesis of SLF:Yb,Tm

**Figure 1:**
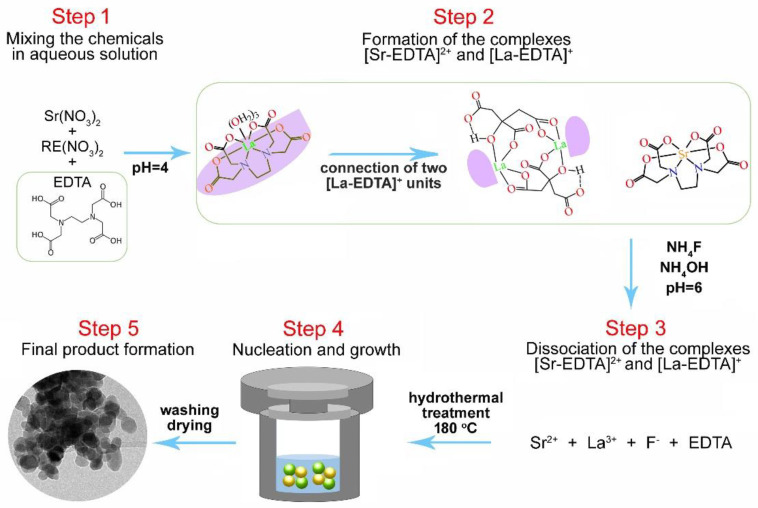
Synthesis of SLF:Yb,Tm nanophosphors using a simple EDTA-assisted hydrothermal method.

Sr_2_La_1-x-y_F_7_:xYb,yTm were synthesized hydrothermally using Sr(NO_3_)_2_, Ln^3+^ nitrates (Ln = La,Yb,Tm), and NH_4_F as precursors and EDTA-2Na as a stabilizing agent (see Figure 1). Typically, for the synthesis of 1 g of the representative sample Sr_2_LaF_7_ co-doped with 20mol% Yb^3+^ and 1 mol% Tm^3+^, all nitrates were weighed according to the stoichiometric ratio (precisely, 0.4762g Sr^3+^-nitrate, 0.3849 g La^3+^-nitrate, 0.1010 g Yb^3+^-nitrate and 0.0050 g Tm^3+^-nitrate) and then dissolved in 12.5 mL deionized water while stirring at room temperature. The above solution was then mixed for 30 min with a transparent solution of 0.4188 g EDTA-2Na in 12.5 mL in water (molar ratio EDTA-2Na:La = 1:1). Following that, a 10 mL aqueous solution containing 0.5001 g of NH_4_F (molar ratio NH_4_F:La = 12:1) was added and vigorously stirred for 1 h, yielding a white complex. Using 400 µL of NH4OH, the pH of the mixture was adjusted to around 6. This mixture was placed in a 100-mL Teflon-lined autoclave and heated in the oven at 180 °C for 20 h. After natural cooling, the final products were centrifuged and washed twice with water, then once with an ethanol:water = 1:1 mixture to remove any possible remnants before drying in an air atmosphere at 80 °C for 4 h. Undoped SLF and SLF phosphors with varying concentrations of Yb^3+^ (x = 10, 15, 20, and 25 mol%) and Tm^3+^ (y = 0.75, 1, 2, and 3 mol%) ions with respect to La^3+^ ions were prepared using the described procedure.

### 2.3. Measurement

X-ray diffraction (XRD) measurements were performed on a Rigaku SmartLab system operating with Cu Kα radiation (30 mA, 40 kV) in the 2θ range from 10° to 90°. Diffraction data were recorded with a step size of 0.02° and a counting time of 1°/min over the investigated 2θ. Results of the structural analysis (unit cell parameters, crystal coherence size, microstrain values, and data fit parameters) were obtained using the built-in PDXL2 software. The microstructure of the samples was characterized by a transmission electron microscope (TEM) Tecnai GF20 operated at 200 kV. The average particle size was calculated using ImageJ software. Diffuse reflectance measurements were performed with the Shimadzu UV-2600 (Shimadzu Corporation, Tokyo, Japan) spectrophotometer equipped with an integrated sphere (ISR-2600), using BaSO_4_ as the standard reference. Luminescence characterization was done using a 980 nm high power (3W) solid state IR laser as an excitation source. Luminescence emissions were recorded using a FHR1000 monochromator (Horiba Jobin Yvon) and an ICCD camera (Horiba Jobin Yvon 3771). All the measurements were performed at room temperature.

## 3. Results and Discussion

### 3.1. XRD Analysis

**Figure 2:**
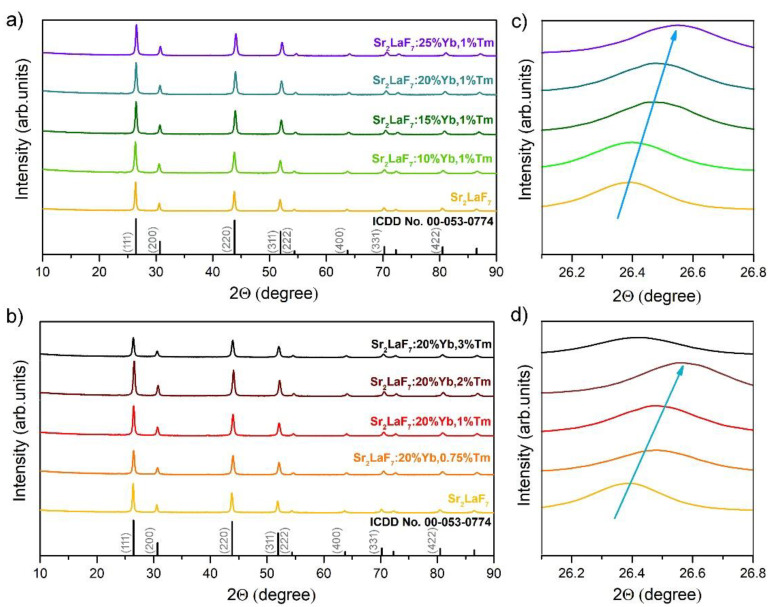
XRD patterns of (**a**) SLF:xYb^3+^,1 mol%Tm^3+^ and (**b**) SLF:20 mol%Yb^3+^,yTm^3+^ nanophosphors. (**c**) The evolution of the (111) diffraction peak magnified from (**a**). (**d**) The evolution of the (111) diffraction peak magnified from (**b**). Arrows indicate changes of the peak position maximum.

The M_x_LnF_2x+3_ fluorides crystallize in a cubic structure with $Fm\bar{3}m$ space group [31]. XRD patterns of SLF:xYb^3+^,1 mol%Tm^3+^ and SLF:20 mol%Yb^3+^,yTm^3+^ nanophosphors are shown in Figure 2a,b, respectively. Despite the addition of Yb^3+^ and Tm^3+^ ions, the main diffraction peaks observed around 2θ = 26.4, 30.7, 43.8, 51.9, 54.4, 63.7, 70.2, and 80.5°, correspond to the main reflections from 111, 200, 220, 311, 222, 400, 331, and 422 crystal planes, respectively, and are well-aligned with the standard data of ICDD No. 00–053–0774 for pure SLF. Diffraction peaks corresponding to other phases and/or impurities were not noticed. The sharp diffraction peaks indicate a good crystallinity of SLF nanophosphors. Table 1 and Table 2 show the results of the structural analysis using whole pattern-fitting (WPF) refinement: crystallite coherence size (CS), microstrain values, unit cell parameters, unit cell volume (CV), and data fit parameters (R_wp_, R_p_, R_e_ and GOF) of SLF:xYb^3+^,1 mol%Tm^3+^ and SLF:20 mol%Yb^3+^,yTm^3+^ nanophosphors. The *CS* of pure SLF is estimated to be 27.1 nm, and the lattice constant *a* is 5.8451 Å (*CV* = 199.70 Å^3^). The influence of Yb^3+^ doping in SLF lattice causes the linear host lattice shrinkage up to *a* = 5.8045 Å, *CV* = 195.57 Å^3^ for the sample SLF:25 mol%Yb^3+^,1 mol%Tm^3+^. This shrinkage could be ascribed to the fact that dopants with smaller ionic radii Yb^3+^ (0.868 Å) and Tm^3+^ (0.880 Å) replace the La^3+^ with larger ionic radii (1.032 Å) in SLF [35]. Tm^3+^ doping in SLF lattice also produces host lattice shrinkage up to *a* = 5.8107 Å, *CV* = 196.2 Å^3^ for the sample SLF:20 mol%Yb^3+^, 2 mol%Tm^3+^. When the concentration of Tm^3+^ ions is increased further, the other doping strategy occurs due to the Tm^3+^ ability to occupy the interstitial sites, leading to crystal lattice expansion (*a* = 5.8288 Å, *CV* = 198.03 Å^3^) [36]. To identify the strategy of Tm^3+^ and Yb^3+^ doping in SLF, the magnified (111) diffraction peak of the samples are shown in Figure 2c,d. With the addition of Yb^3+^ ions, the position of the (111) diffraction peak consistently shifts to higher degree values, and the shift becomes more notable with increasing dopant concentration (Figure 2c). Tm^3+^ doping in SLF has the same behavior at concentrations equal to or less than 2 mol%, while further addition of Tm^3+^ shifts the diffraction peak to lower degree values (Figure 2d). These findings confirm that both Yb^3+^ and Tm^3+^ (equal or less than 2 mol%) were successfully inserted into SLF by occupying La^3+^ sites.

### 3.2. Morphology Analysis

An assisted EDTA hydrothermal method was used to create SLF:Yb, Tm nanophosphors. EDTA is an efficient complexing agent for Ln^3+^ ions, with chelation constants (logK_1_) of 19.51 and 15.50 for Yb^3+^ and La^3+^ ions, respectively [31]. Due to the ability to improve crystalline seed dispersibility by forming [Sr-EDTA]^2+^ and [La-EDTA]^+^ complexes after mixing all of the chemicals, EDTA prevented SLF particle aggregation during the subsequent hydrothermal treatment. On the other hand, [La-EDTA]^+^ cations could be absorbed on the surfaces of SLF particles, limiting their further growth into large particles, and also increasing their stability [36,37].

**Figure 3:**
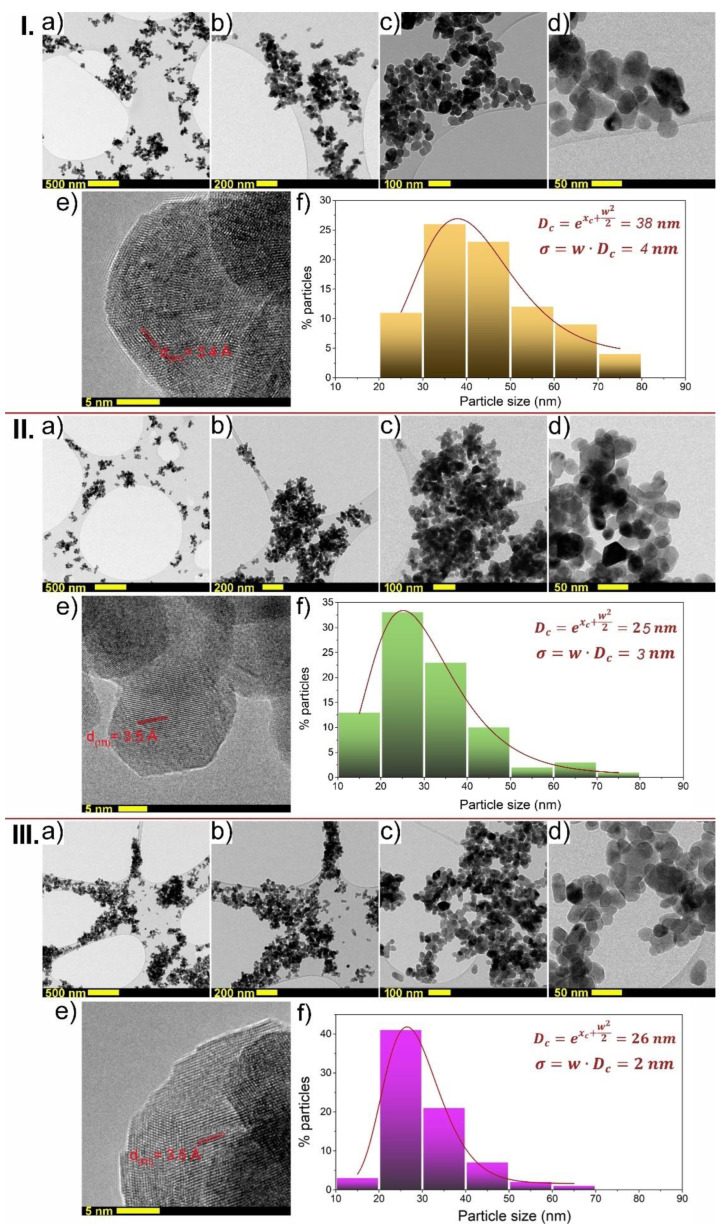
**I. Undoped SLF:** (**a**–**d**) TEM images of hydrothermally synthesized SLF, (**e**) HRTEM image of SLF, (**f**) particle size distribution of SLF. **II. SLF:10Yb,1Tm:** (**a**–**d**) TEM images (**e**) HRTEM image, (**f**) particle size distribution. **III. SLF:25Yb,1Tm:** (**a**–**d**) TEM images, (**e**) HRTEM image, (**f**) particle size distribution.

TEM images of undoped SLF with different magnifications together with particle size distribution histogram are shown in Figure 3I. Nanoparticles show a similar, quasi-spherical shape as well as a high degree of crystallinity. HRTEM image of SLF phosphors (Figure 3Ie) shows that the measured *d*-spacing is around 3.4 Å, that corresponds to the (111) lattice plane of SLF, which agrees to the previous XRD data. The half-displayed particles were not considered when calculating the average particle size, and the histogram was fitted with a log-normal distribution. The average crystalline size of nanoparticles, considering around 120 particles, was estimated to be 38 ± 4 nm (see Figure 3If). The influence of Yb^3+^ and Tm^3+^ co-doping on the morphology of SLF samples can be observed by comparing features in Figure 3I, 3II and 3III, respectively. The average particle size of SLF nanophosphor doped with 10 mol% Yb^3+^ (Figure 3IIa–f) and SLF doped with 25 mol% Yb^3+^ (Figure 3IIIa–f) ions and a fixed concentration level of 1 mol% Tm^3+^ was calculated to be 25 ± 3 nm and 26 ± 2 nm, respectively. Therefore, the average particle size of SLF was reduced by doping from 38 to around 25 nm, which is well-aligned with the previous XRD analysis. HRTEM images of both SLF:10Yb,1Tm (Figure 3IIe) and SLF:25Yb,1Tm phosphors (Figure 3IIIe) show that the measured d-spacing is around 3.5 Å, which also corresponds to the (111) lattice plane of SLF. As previously explained, the addition of Yb^3+^/Tm^3+^ ions has a slight impact on the lattice constant when compared to undoped SLF because dopants with smaller ionic radii Yb^3+^ and Tm^3+^ replace the La^3+^ with larger ionic radii. The average particle size of SLF nanophosphor, on the other hand, is strongly influenced by the concentrations of Yb^3+^ and Tm^3+^.

### 3.3. Spectroscopic Properties

**Figure 4:**
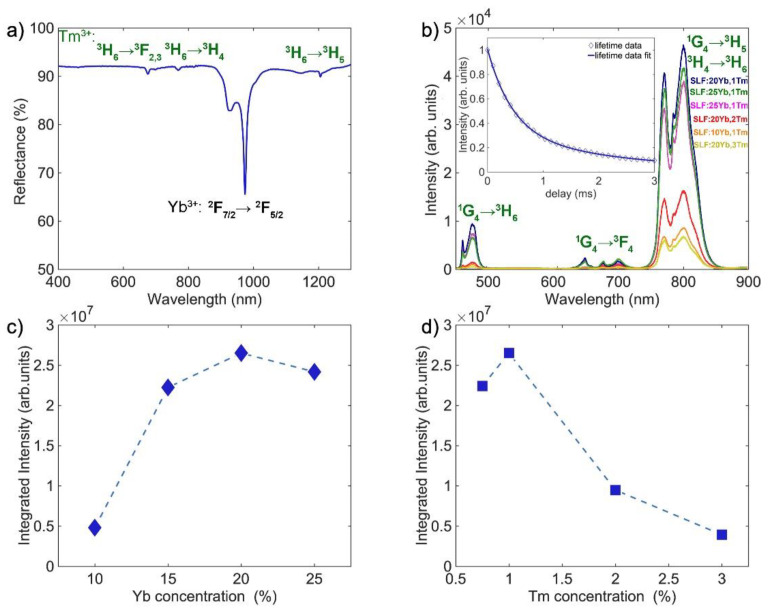
(**a**) Diffuse reflectance spectra of representative SLF:20Yb,1Tm nanophosphor. (**b**) PL spectra of SLF: xYb, yTm nanophosphors measured upon 980 nm excitation (Inset: UC lifetime profiles of Tm^3+^ (^3^H_4_ → ^3^H_6_ transition) of representative SLF:20Yb,1Tm). (**c**) Yb^3+^ concentration dependence of integrated UC emission intensity of SLF nanophosphor. (**d**) Tm^3+^ concentration dependence of integrated UC emission intensity of SLF nanophosphor.

Figure 4a shows the room temperature diffuse reflectance spectra of a representative SLF:20Yb,1Tm sample in the 400–1300 nm wavelength range with typical optical features of Yb^3+^ and Tm^3+^ ions [38]. The absorption peaks of Yb^3+^ ions appear in the 885–1060 nm wavelength range due to electronic transitions from ^2^F_7/2_ → ^2^F_5/2_, with the highest intensity around 980 nm. In the case of Tm^3+^ ions, three major electronic transitions are involved: ^3^H_6_ → ^3^F_2,3_, ^3^H_6_ → ^3^H_4_, and ^3^H_6_ → ^3^H_5_, which correspond to absorption peaks at 677 nm, 770 nm, and 1206 nm, respectively.

The room temperature emission spectra of a representative SLF:20Yb,1Tm nanophosphor in the 450–900 nm wavelength range are shown in Figure 4b. In a typical multiphoton UC process, Yb^3+^ absorbs NIR radiation at around 980 nm which causes electron excitation from ^2^F_7/2_ to ^2^F_5/2_ energy level. Then, the Tm^3+^ is excited via cross-relaxation and energy transfer from excited Yb^3+^. The deexcitation from multiple Tm^3+^ excited levels provide emissions that cover UV–VIS–NIR spectra. The observed emission peaks, which occur at wavelengths ranging from 455 to 500 nm, 625 to 720 nm, and 750 to 850 nm, are attributed to the transitions from the ^1^G_4_ → ^3^H_6_, ^1^G_4_ → ^3^F_4_, and ^1^G_4_ → ^3^H_5_ / ^3^H_4_ → ^3^H_6_ of excited Tm^3+^ ions, respectively. The PL decay curve at room temperature is shown in the inset of Figure 4b. The average emission time (τ_av_), calculated based on the double exponential model, was used as a measurement of PL lifetime (τ). Through the fit of our experimental data to the double exponential model, the two values of τ_1_ and τ_2_ are obtained:

(1) $$I\left(t\right)=A_{1}e^{-\frac{t}{\tau_{1}}}+A_{2}e^{-\frac{t}{\tau_{2}}}+bg.$$

where, *A*_1_ and *A*_2_ are arbitrary constants (magnitudes of short and long decay components), and *bg* is a background correction. Because the measured signal (𝐼 (𝑡)) at delayed time 𝑡_𝑑_ is proportional to the number of excited states at the moment 𝑡_𝑑_, the simple weighted average formula is used to calculate 𝜏_av_:

(2) $$\tau_{av}=\frac{A_{1}\tau_{1}+A_{2}\tau_{2}}{A_{1}+A_{2}}.$$

The PL lifetime of Tm^3+^ (^3^H_4_ → ^3^H_6_ transition) in a representative SLF:20Yb,1Tm nanophosphor was estimated to be 1.05 ms. Table 3 summarizes the arbitrary constants, background correction, two values of PL lifetime (τ_1_ and τ_2_), and average PL lifetime of the representative SLF:20Yb,1Tm nanophosphor. The deviation of the ^3^H_4_ → ^3^H_6_ emission decay from the single exponentiality indicates that energy back-transfer to Yb^3+^ occurs from ^3^H_4_ level. This can be further investigated by measuring the variation in lifetimes of Yb^3+ 2^F_5/2_ emission for different Tm^3+^ and Yb^3+^ concentrations.

The UC emission intensity relates to both Yb^3+^ and Tm^3+^ concentrations. Figure 4c presents the dependence of the integrated UC emission intensity of SLF with different concentrations of Yb^3+^ (x = 10, 15, 20, and 25 mol%) and a fixed Tm^3+^ concentration (1 mol%). With increasing Yb^3+^ concentration, the NIR emission intensity band increases, reaching a maximum value at 20 mol% of Yb^3+^. Similarly, when Yb^3+^ concentration is fixed at 20 mol%, the NIR emission of SLF monitored at different concentrations of Tm^3+^ (x = 0.75, 1, 2, and 3 mol%) has the highest intensity for 1 mol% Tm^3+^, as shown in Figure 4d. When the Tm^3+^ doping concentration is equal to or greater than 2, the emission intensity decreases gradually due to the concentration quenching. In contrast to the emission intensity, the shape and characteristic peak position of the UC emission spectra have not changed. For the Yb^3+^ and Tm^3+^ co-doped phosphors, blue (^1^G_4_ → ^3^H_6_) and deep-red (^1^G_4_ → ^3^F_4_) emissions have been widely investigated [27,38,39,40], while the efficient NIR-to-NIR Yb^3+^/Tm^3+^ UC emission in ultrasmall SLF nanophosphor has received far less attention. Therefore, ultrasmall SLF nanoparticles with intense emission around 800 nm are promising candidates as fluorescent labels in bioimaging and security applications.

## 4. Conclusions

In conclusion, ultrasmall SLF:Yb^3+^/Tm^3+^ nanoparticles were produced using a straightforward hydrothermal process at a variety of doping doses. With Yb^3+^ and Tm^3+^ ions present, the lattice constant and average particle size of SLF are both decreased from 38 nm to roughly 25 nm. At room temperature, Yb^3+^ and Tm^3+^ concentrations have a significant impact on the PL emission properties. When excited with a 980 nm high power (3W) solid state IR laser, these ultrasmall nanoparticles show simultaneous three-color (blue-green, deep-red, and NIR) UC emissions in the 450–900 nm wavelength range. The blue-green and deep-red emission bands are weak, while the NIR emission band is strong, which is beneficial for imaging biological tissues. Furthermore, the PL lifetime of Tm^3+^ (^3^H_4_ → ^3^H_6_ transition) in a representative SLF:20Yb,1Tm nanophosphor was estimated to be 1.05 ms. These findings also suggest that SLF:Yb,Tm could be a useful fluorescent marker in the development of latent fingerprints. Our future work will concentrate on the dual-mode fluorescent development of latent fingerprints using both NIR-to-VIS and NIR-to-NIR processes to achieve double fluorescent images in dark and bright fields, as well as additional contrast and sensitivity analysis of fingerprints or fingerprint residuals deposited on a variety of substrates. Furthermore, the proposed NIR-to-NIR UC mechanism of ultrasmall Yb^3+^/Tm^3+^ co-doped SLF nanophosphors could be a useful tool in security applications.

## Figures and Tables

**Table 1:** Results of the structural analysis by using WPF refinement of SLF:xYb^3+^, 1 mol%Tm^3+^ nanophosphors.

	SLF	SLF:10Yb,1Tm	SLF:15Yb,1Tm	SLF:20Yb,1 Tm	SLF:25Yb,1Tm
*CS* (nm)	27.1	23.5	25	25.4	24.4
Strain	0.144	0.254	0.254	0.272	0.258
* R_wp_	6.00	4.74	4.58	4.33	4.09
** R_p_	4.62	3.79	3.51	3.35	3.28
*** R_e_	3.42	3.48	3.37	3.37	3.28
GOF	1.7569	1.3597	1.3597	1.2843	1.2484
*a* = *b* = *c* (Å)	5.8451	5.8372	5.8190	5.8115	5.8045
*CV* (Å^3^)	199.70	198.89	197.04	196.27	195.57

* R_wp_—the weighted profile factor; ** R_p_—the profile factor; *** R_e_—the expected weighted profile factor; GOF—the goodness of fit.

**Table 2:** Results of the structural analysis by using WPF refinement of SLF:20 mol%Yb^3+^,yTm^3+^ nanophosphors.

	SLF	SLF:20Yb,0.75Tm	SLF:20Yb,1Tm	SLF:20Yb,2Tm	SLF:20Yb,3Tm
*CS* (nm)	27.1	22.6	25.4	21.9	17.8
Strain	0.144	0.211	0.272	0.185	0.02
* R_wp_	6.00	4.12	4.33	4.91	5.97
** R_p_	4.62	3.14	3.35	3.90	4.41
*** R_e_	3.42	3.56	3.37	3.33	3.39
GOF	1.7569	1.1589	1.2843	1.4747	1.7607
*a* = *b* = *c* (Å)	5.8451	5.8129	5.8115	5.8107	5.8288
*CV* (Å^3^)	199.70	196.42	196.27	196.20	198.03

* R_wp_—the weighted profile factor; ** R_p_—the profile factor; *** R_e_–the expected weighted profile factor; GOF—the goodness of fit.

**Table 3:** Summary of the different parameters used to calculate PL lifetime of the representative SLF:20Yb,1Tm nanophosphor.

$A_{1}$	$\tau_{1}\;\left(ms\right)$	$A_{2}$	$\tau_{2}\;\left(ms\right)$	bg	τ (ms)
0.6887	0.4503	0.3163	2.3607	0.0030	1.0516

## Data Availability

The data presented in this study are available on request from the corresponding author.
